# High‐Performance and Reversible Bitter Taste Sensing Using Aptamer‐Functionalized Graphene Channel for Real‐Time Electronic Tongue

**DOI:** 10.1002/exp2.70232

**Published:** 2026-10-27

**Authors:** Hyuk Jin Kim, Haesoo Hwang, Jun Uh Hyun, Sungwoo Park, Sohyeon Park, Yeong Jae Kim, Hyeon‐Ji Lee, Hee Ryeong Kwon, Seon Ju Park, Gi Baek Nam, Min Gyeong Choe, Byungsoo Kim, Soo‐Yeon Cho, Je Oh Choi, Jong‐Hoon Kang, Dong Sung Kim, Jun Min Suh, Youngeun Kim, Ho Won Jang

**Affiliations:** ^1^ Department of Materials Science and Engineering, Research Institute of Advanced Materials Seoul National University Seoul Republic of Korea; ^2^ Department of Electrical and Computer Engineering University of Virginia Charlottesville Virginia USA; ^3^ School of Chemical Engineering Sungkyunkwan University (SKKU) Suwon Republic of Korea; ^4^ Department of Materials Science and Engineering Pohang University of Science and Technology (POSTECH) Pohang Republic of Korea; ^5^ Department of Mechanical Engineering Pohang University of Science and Technology (POSTECH) Pohang Republic of Korea; ^6^ School of Transdisciplinary Innovations Seoul National University Seoul Republic of Korea; ^7^ Advanced Institute of Convergence Technology Seoul National University Suwon Republic of Korea

**Keywords:** aptamer, field‐effect transistor, graphene, quinine, taste sensor

## Abstract

Dynamic and real‐time sensing of taste is increasingly important for food safety and pharmaceutical quality control, yet the development of advanced sensors for each taste, especially those for bitterness, remains limited. The challenge has been lying on developing suitable receptors for bitter compounds that are capable of reversible operation. Here, we present a graphene‐based taste sensor that integrates a quinine‐specific aptamer, enabling selective molecular recognition with high sensitivity and operational stability. Especially, unlike previous sensors, which were irreversible or required regeneration steps, our design achieves fully reversible quinine‐specific capture and release. Combined with previously developed taste sensors by the authors, graphene‐based electronic tongue was demonstrated to differentiate four different taste compounds and even complex beverages. This work highlights the strong potential of the graphene‐based sensors with aptamer receptors as reliable and tunable platforms for advanced taste sensing and real‐time analysis.

## Graphical abstract

A graphene sensor functionalized with a DNA aptamer selectively detects quinine, a bitter compound, and recovers for repeated measurements. Combined with other taste sensors and wireless electronics, it distinguishes beverage taste profiles, demonstrating an electronic tongue with potential for beverage quality assessment.

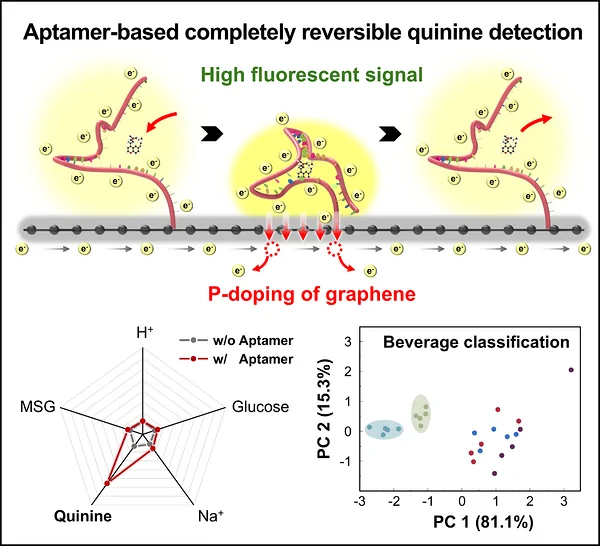

## 1 Introduction

The accurate detection of bitter compounds is essential for food safety, pharmaceutical quality control, and beverage certification, as excessive bitterness can indicate contamination or improper formulation. Quinine serves as the reference standard for bitterness and is strictly regulated in food due to its potential adverse physiological effects. However, the reliable and selective detection of quinine in complex matrices remains a persistent challenge due to the structural diversity of bitterants, the presence of interfering substances, and the lack of robust quinine‐selective molecular receptors that can provide high specificity in complex matrices [1, 2]. Publication trends for taste sensors are summarized in Figure S1.

Conventional taste sensors, including those based on lipid membranes or polymeric films, have been widely explored for gustatory signal detection and classification. However, these systems suffer low selectivity as well as high cross‐reactivity arising from the structural complexity and the similarity of bitter compounds such as quinine [3, 4]. More importantly, they exhibit poor reversibility due to analyte‐receptor binding that is difficult to release once analytes are strongly bound. This limited dissociation leads to a baseline drift, memory effects, and the need for frequent recalibration, particularly after repeated measurements.

In this context, graphene‐based field‐effect transistors (FETs) have recently emerged as promising candidates for reversible chemical sensors taking advantage of their surface properties as well as their exceptional electrical properties, including high carrier mobility and sensitive modulation upon surface charge variations [5, 6, 7]. As a channel material, graphene offers strong intrinsic advantages: its surface has very few dangling bonds, which minimizes irreversible chemical interactions and helps preserve sensor stability and reusability. Moreover, its atomically thin, two‑dimensional structure and delocalized π‑electron system make it extremely responsive to changes in the local electronic environment. Any interaction or perturbation at the graphene surface is barely screened and can influence the conductance of the entire channel [6]. These characteristics make graphene particularly well suited for non‑chemical‑binding, non‑specific sensing mechanisms, where even subtle variations in surface charge are efficiently transduced into measurable electrical signals. Although graphene's non‑specific detection makes it highly effective for detecting a wide range of analytes, its pristine form inherently lacks molecular selectivity, which can lead to cross‑reactivity with chemically similar species in complex real‑world samples [5]. Therefore, several approaches have been explored to enhance the selectivity, including analyte‐receptor specific binding reaction, but they still face many challenges like slow and incomplete recovery.

In this study, MNS‐4.1 aptamer (Figure S2), a synthetic DNA sequence with tunable affinity, has been integrated with graphene to provide target selectivity with complete recovery. Upon interacting with specific molecules, the aptamer undergoes a reversible conformational rearrangement that redistributes its negatively charged phosphate backbone. This structural change modulates the local electrostatic environment and, in turn, the charge carrier distribution within the graphene channel, producing a measurable shift in its electrical characteristics [1, 8]. Under the microfluidic taste sensor platform, the real‐time quinine monitoring was demonstrated with high sensitivity, operational stability, and clear selectivity over common interferents, with reversible operation [9]. Combined with previously developed taste sensors by the authors, the graphene‐based electronic tongue was demonstrated to differentiate four different taste compounds and even complex beverages [6, 10]. Principal component analysis further validates its ability to discriminate complex taste profiles in real‐world environments [11, 12]. This work establishes a robust and tunable framework for next‐generation taste sensors, bridging biomolecular specificity and nanomaterial‐enabled signal transduction, advancing bitterness detection toward practical applications [5, 7].

## 2 Results and Discussion

### 2.1 Reversible Quinine Recognition Confirmed on an Aptamer‐Functionalized Graphene Sensing Interface

Figure 1a shows a schematic illustration of MNS‐4.1 aptamer‐functionalized graphene and its electron transport mechanism during quinine sensing. On the graphene surface, quinine binding causes a structural change of MNS‐4.1 that modifies the local charge environment. For quinine detection, this conformational shift changes the electron transport characteristics of the graphene channel, which are eventually transduced into a measurable electrical signal [13, 14]. Previous studies on the MNS‐4.1 aptamer characterized its binding sequence and demonstrated its functionality primarily through complex fluorescence‐based detection schemes, without employing graphene substrates [8, 15]. Building on these reports, we adapted the aptamer sequence as a molecular recognition element directly integrated onto a graphene‐based FET. Unlike prior fluorescence‐focused approaches, our strategy enables real‐time, reversible, and highly sensitive sensing on a graphene platform. This adaptation illustrates how aptamers previously validated in optical assays can be transformed into practical electronic sensor devices, broadening their applicability and underscoring the versatility of graphene‐based systems for selective small‐molecule detection.

**FIGURE 1:**
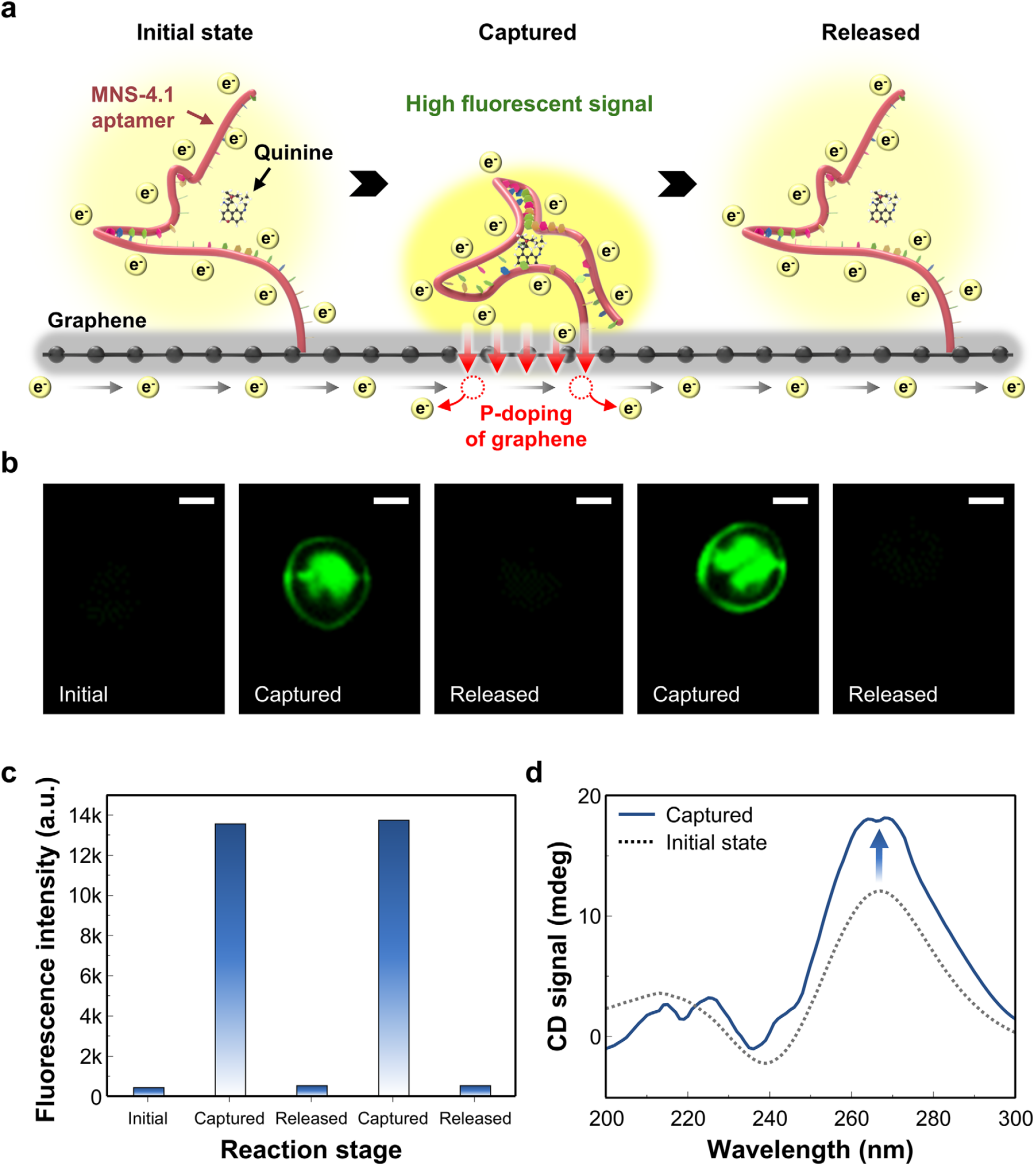
Detection mechanism and molecular recognition process of the aptamer‐functionalized graphene quinine sensor. (a) Schematic illustration of the detection mechanism using MNS‐4.1 aptamer, showing state transitions (initial, captured, and released) and corresponding fluorescence changes induced by p‐doping of graphene. (b) Sequential fluorescence images of the aptamer‐functionalized graphene sensor in each state, monitored using Eva Green dye, demonstrating fluorescence changes upon transition between initial, captured, and released states. All scale bars, 1 mm. (c) Quantitative fluorescence intensity measurement for each state, demonstrating the reversibility of aptamer state transitions. (d) CD spectra of the aptamer in the initial state (dotted line) and captured state (solid line), demonstrating conformational rearrangements of the aptamer upon transition to the captured state.

To assess the reversibility of target binding, fluorescence‐based monitoring using Eva Green dye was performed through sequential cycles of quinine exposure and washing (Figure 1b). Eva Green is a DNA‐binding dye that fluoresces strongly upon intercalation with double‐stranded or structured regions of nucleic acids (Figure S3). We hypothesized that quinine binding would induce a conformational change in the aptamer, increasing intra‐strand base pairing and thus enhancing Eva Green fluorescence. The aptamer‐functionalized graphene substrate exhibited strong fluorescence signals in the captured state (after quinine exposure), which diminished in the following released state (after washing steps), indicating efficient and reversible target interaction [16, 17, 18]. This reversibility was further supported by quantitative fluorescence analysis (Figure 1c), which showed that intensity values returned to baseline levels after each washing cycle (initial and released states). We further validated the sensing mechanism by testing a ‘scrambled aptamer’ control, which shares the identical nucleotide composition (and charge density) with the native MNS‐4.1 but lacks its specific secondary structure. As shown in Figure S4, both the poly‐T and scrambled sequences yielded negligible responses to quinine. This definitively proves that the electronic signal arises from the target‐induced conformational rearrangement rather than non‐specific electrostatic adsorption. To investigate the structural basis of quinine recognition, circular dichroism (CD) spectroscopy was conducted (Figure 1d) [8]. The CD spectra exhibited a marked increase in ellipticity between 260 and 280 nm (blue solid line, captured state) after quinine binding, compared to the unbound aptamer (initial state, gray dotted line). This spectral shift reflects a conformational rearrangement in the MNS‐4.1 aptamer, particularly in stem‐loop regions, enabling specific and selective molecular recognition of the quinine molecule [19, 20].

### 2.2 Structural and Surface Characterization of Sensor Components

Computational prediction of the MNS‐4.1 aptamer's secondary structure (Figure 2a) was employed to investigate its conformational changes upon quinine binding. In the absence of the target, the aptamer adopts a relatively simple stem‐loop configuration, characterized by a large circular loop structure and a small hairpin region. This unbound conformation exhibits a calculated free energy of −3.3 kcal/mol, with equilibrium probability mapping (visualized via a color gradient) indicating heterogeneous structural stability across different regions. Upon quinine binding, the aptamer is predicted to undergo a substantial conformational rearrangement, transitioning into a more complex architecture comprising a multi‐stem‐loop structure with enhanced base pairing. This target‐bound state demonstrates a significantly lower free energy of −4.82 kcal/mol, yielding a ΔG of −1.52 kcal/mol that suggests improved thermodynamic stability [21, 22, 23].

**FIGURE 2:**
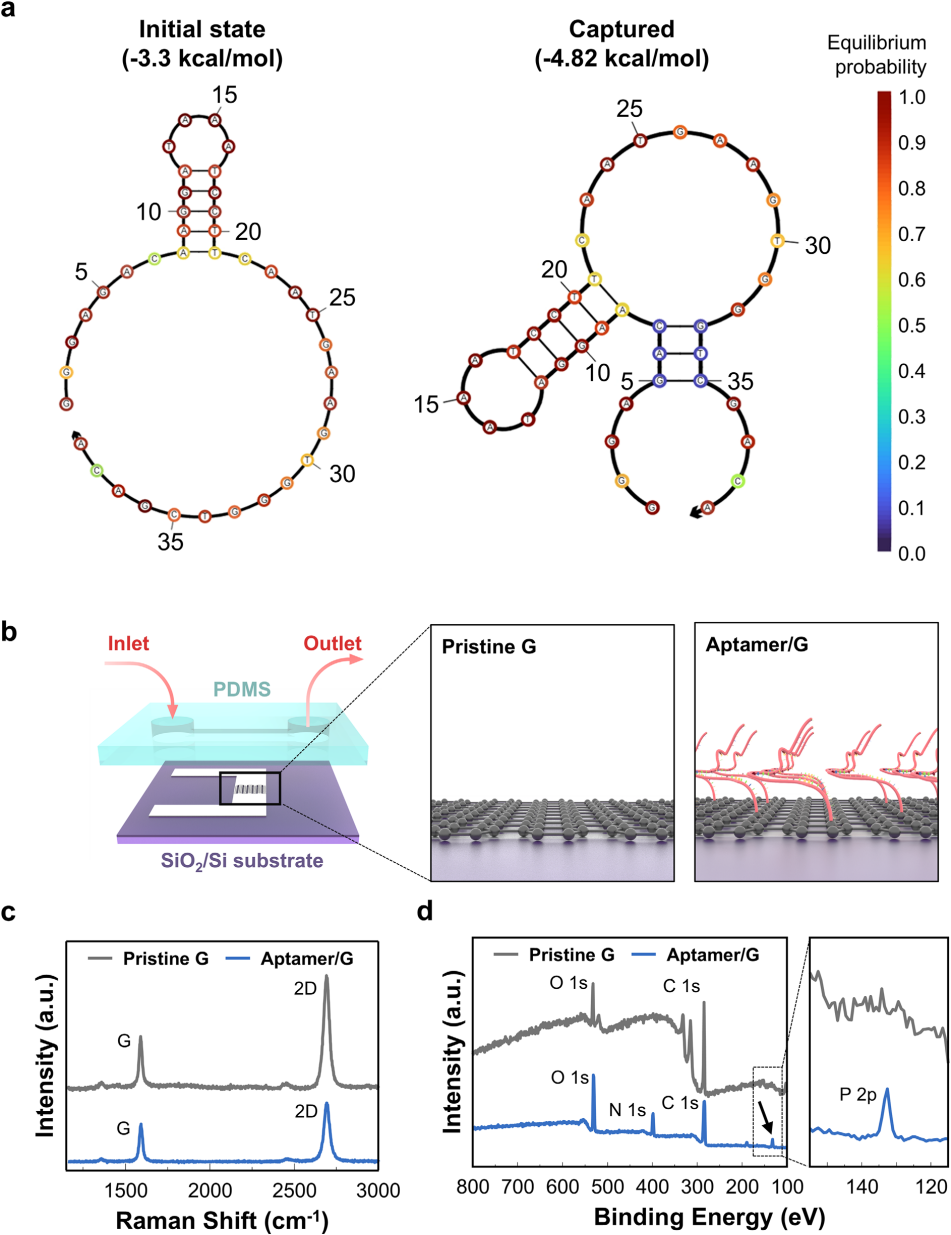
Structural transitions of the MNS‐4.1 aptamer and surface characterization of the aptamer‐functionalized graphene sensor system. (a) Simulation‐predicted secondary structures of the MNS‐4.1 aptamer in the initial state (left, free energy −3.3 kcal/mol) and captured state (right, free energy −4.82 kcal/mol). Equilibrium probability is represented by color mapping, illustrating structural rearrangement and increased stability in the captured state. (b) Schematic cross‐sectional representation of the device architecture and comparison of Pristine G (graphene) and Aptamer/G (aptamer‐functionalized graphene) surfaces. (c) Raman spectra and (d) XPS analyses of Pristine G and Aptamer/G surfaces, validating aptamer immobilization and the corresponding changes in surface elemental composition.

This structural change directly correlates with the sensing mechanism of the aptamer‐functionalized graphene taste sensor. The energy‐minimized conformation of the quinine‐bound aptamer suggests that target recognition promotes additional base pairing interactions and tertiary contacts, stabilizing the overall aptamer structure. This conformational change redistributes the negatively charged phosphate backbone relative to the graphene surface, thereby effectively modulating charge carrier density and electrical conductivity through the graphene channel. The improved thermodynamic stability of the aptamer‐quinine complex compared to the unbound state provides a quantitative basis for the high binding affinity and selectivity observed in our experimental measurements [8]. This relationship between structure and function provides a rational system for designing aptamer‐functionalized taste sensors with adjustable selectivity profiles for diverse sensing applications. Additionally, the observed shift in free energy is relatively small, which implies that the aptamer and quinine have a binding relationship that is strong enough to provide precise recognition while weak enough for reversible association and dissociation. In particular, the low‐probability regions indicated by blue coloring in the captured state aptamer structure (Figure 2a) correspond to nucleotides involved in flexible base interactions, which facilitate rapid conformational rearrangement. This localized instability within the quinine‐bound complex plays a crucial role in promoting aptamer reversibility, thereby enabling prompt dissociation and regeneration required for reusable sensing cycles. Sensor applications that need to repeatedly detect and regenerate targets benefit greatly from this binding profile [9, 13].

Based on the mechanism, an aptamer‐functionalized graphene taste sensor was fabricated with a PDMS (polydimethylsiloxane) microfluidic channel as shown in Figure 2b. The device is constructed on a SiO_2_/Si substrate, and the PDMS microfluidic channel is aligned for controlled sample delivery. The microfluidic channel, with its gentle flow, allows dynamic, reversible solution exchange, facilitating real‐time monitoring of aptamer conformational dynamics and ensuring stable baseline signals [20, 21]. The structure and arrangement of the graphene and aptamer‐functionalized graphene channels are verified by optical microscope (OM) images, verifying that the microfluidic system and the sensing platform are properly fabricated for sensing experiments (Figure S5) [6, 10]. The taste sensor platform, as depicted in Figure S6, adopts a bottom‐gate FET structure, where the graphene layer functions as the conducting channel between the source and drain electrodes. The detection site on the graphene channel is functionalized with the aptamer via non‐covalent π‐π stacking, enabling specific recognition of quinine.

The morphology and crystallinity of the graphene layer were characterized using transmission electron microscopy (TEM) and selected area electron diffraction (SAED), as shown in Figure S7 [11, 12]. While the SAED pattern provides localized information on the crystalline orientation, the overall lattice quality and macroscopic uniformity of the monolayer graphene were further verified through Raman spectroscopic mapping. As presented in Figure S8, the Raman mapping results, including the intensity ratio histogram of I_2D_/I_G_, demonstrate the presence of a high‐quality monolayer graphene lattice across a large area. This spatial uniformity in lattice features is crucial for ensuring reproducible electrical performance across multiple sensor devices. Raman spectroscopy results (Figure 2c) reveal distinct changes in the electronic environment of graphene upon aptamer functionalization. After aptamer modification, the Raman spectra exhibit a shift in the G peak position along with a decrease in the 2D/G intensity ratio. These spectral changes indicate that aptamer immobilization alters the electron density distribution on the graphene surface, consistent with the influence of the negatively charged phosphate backbone of the DNA. This confirms successful non‐covalent functionalization and demonstrates that aptamer attachment induces significant modulation of the local electronic structure of graphene [24, 25, 26]. X‐ray photoelectron spectroscopy (XPS) analysis provided insight into the chemical composition and bonding states of the graphene and aptamer‐functionalized graphene samples (Figure 2d). The appearance of additional N 1s (399 eV) and P 2p peaks (132.6 eV) in the aptamer‐functionalized graphene spectra confirms the successful immobilization of the DNA aptamer on the graphene surface. Therefore, these results prove the effective fabrication, functionalization, and operation of the aptamer‐functionalized graphene taste sensor, establishing its potential as a highly selective and reversible sensor device for quinine detection [27]. The sensor performance is intrinsically governed by the quality of the aptamer interface, specifically immobilization efficiency, orientation, and surface density. First, the robust immobilization via π‐π stacking was evidenced not only by the spectroscopic signatures (Raman and XPS) but also by the exceptional long‐term stability over 290 days (to be discussed below), indicating that the non‐covalent anchoring is sufficiently strong to withstand microfluidic shear forces [28]. Second, regarding molecular orientation, the aptamers are postulated to adopt an ‘anchor‐and‐loop’ configuration [13]. While aromatic nucleobases stack on the graphene surface serving as anchors, the negatively charged phosphate backbone and the recognition loop protrude into the Debye length region, maintaining accessibility for quinine binding. Finally, the surface density was optimized to minimize steric hindrance. The high sensitivity and rapid response kinetics observed in this study suggest that the aptamers are immobilized at a density that provides sufficient free volume for target‐induced conformational folding, avoiding the overcrowding effects often observed in high‐density arrays [29].

### 2.3 Electronic Transport Characteristics and Concentration‐Responsive Sensing Mechanism of Aptamer‐Functionalized Graphene Field‐Effect Transistors

Transfer characteristics of the aptamer‐functionalized graphene system exhibit a noticeable positive shift in the Dirac point voltage with increasing quinine concentration (1–5 mM), consistent with enhanced p‐type carrier doping induced by target binding [13]. This electronic transduction mechanism can be attributed to the conformational rearrangement of the aptamer upon quinine binding, which modulates the local electrostatic environment at the graphene‐electrolyte interface and induces charge carrier redistribution within the graphene channel [30, 31]. The aptamer's negatively charged phosphate backbone is repositioned closer to the graphene surface as a result of its conformational change during quinine binding, which increases electrostatic interaction and induces p‐type doping in the graphene channel. To further elucidate the electrostatic gating mechanism, we considered the Debye screening effect, which dictates the effective sensing distance in an electrolyte environment. The Debye length (*λ_D_*) represents the distance over which the electric field from surface charges is screened by mobile ions in the solution, calculated by the equation [13]:

(1) $$\lambda_{D}=\sqrt{\frac{\varepsilon_{0}\varepsilon_{r}k_{B}T}{2N_{A}e^{2}I}}$$

Under the experimental conditions, *λ_D_* is estimated to be typically less than 1 nm. In the unbound state, the MNS‐4.1 aptamer exhibits a relatively loose conformation, positioning a significant portion of its negatively charged phosphate backbone outside this screening limit. However, upon specific binding with quinine, the aptamer undergoes a structural contraction into a compact fold (as predicted in Figure 2a). This conformational change effectively translocates the negative charges from the screened region within the Debye length, thereby significantly modulating the surface potential and inducing p‐type doping in the graphene channel [9, 13].

This spatial rearrangement of negative charges at the graphene‐electrolyte interface directly modulates the carrier concentration and contributes to the observed positive shift in gate‐dependent conductivity. The aptamer's three‐dimensional structural change upon quinine binding offers molecular recognition specificity, as demonstrated by the negligible response observed in pristine graphene (Figure 3a) compared to the significant Dirac point shifts in the aptamer‐functionalized device that vary with concentration (Figure 3b). Therefore, the resulting sensor exhibits high selectivity, sensitivity, and excellent signal reversibility upon analyte removal, establishing its robustness for real‐time, label‐free bitterant detection [13, 32]. As reliable sensing requires a linear correlation between analyte concentration and sensor response, the gate voltage was set to a specific value to ensure consistent sensor linearity. Further measurements were all conducted at a gate voltage of −0.2 V. This specific operating bias was strategically selected to exploit the highly linear response region of the device, effectively translating the target‐induced electrostatic gating effect into a predictable electrical output. This approach ensures that despite the complex nature of charge transport in graphene FETs, the sensor provides reliable and linear concentration‐dependent signals, which is essential for accurate quantitative determination in practical applications.

**FIGURE 3:**
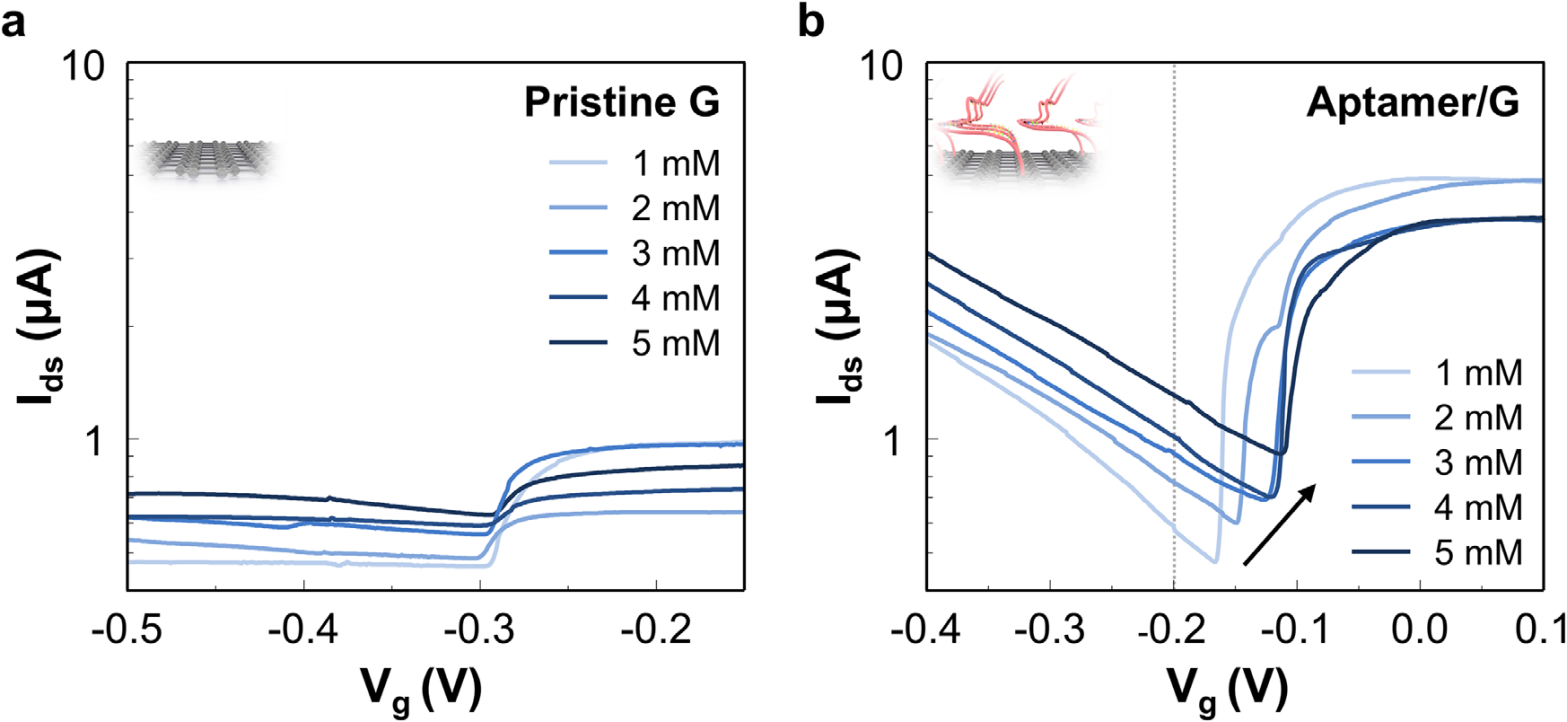
Electrical properties of pristine and aptamer‐functionalized graphene FETs upon analyte exposure. Transfer characteristics of (a) Pristine G (graphene) and (b) Aptamer/G (aptamer‐functionalized graphene) FET devices measured upon exposure to increasing concentrations of quinine. Distinct gate voltage shifts are observed for the Aptamer/G device, reflecting sensitive and concentration‐dependent modulation of electronic properties induced by selective analyte recognition.

To evaluate the sensing performance of our aptamer‐functionalized graphene taste sensor, we conducted extensive analysis comparing pristine graphene and aptamer‐functionalized graphene devices under various experimental conditions (Figure 4). The repetitive response characteristics of the graphene‐based taste sensor upon exposure to 5 mM quinine solution revealed little signal changes with low response magnitudes (Figure 4a). When tested against different quinine concentrations (1–5 mM), the graphene sensor exhibited only slight variations in response as a function of quinine level (Figure 4b), indicating limited interaction between pristine graphene and quinine molecules [33, 34]. The detection limits of both sensor types were rigorously assessed by testing quinine concentrations, ranging from low micromolar to millimolar levels. The graphene‐based taste sensor exhibited small but detectable responses at millimolar concentrations (Figure 4c). The aptamer‐functionalized graphene taste sensor, on the other hand, displayed considerably improved sensing capabilities. The aptamer‐functionalized graphene taste sensor generated continuous, highly reproducible response peaks with a much larger amplitude (Figure 4d) after being repeatedly exposed to 5 mM quinine solution. This dramatic enhancement can be attributed to the specific molecular recognition ability of the MNS‐4.1 aptamer. The aptamer‐functionalized graphene taste sensor exhibited a high dynamic range across the tested concentration range (1–5 mM), with distinct and quantitative response profiles that varied consistently with quinine concentration (Figure 4e) [35, 36].

**FIGURE 4:**
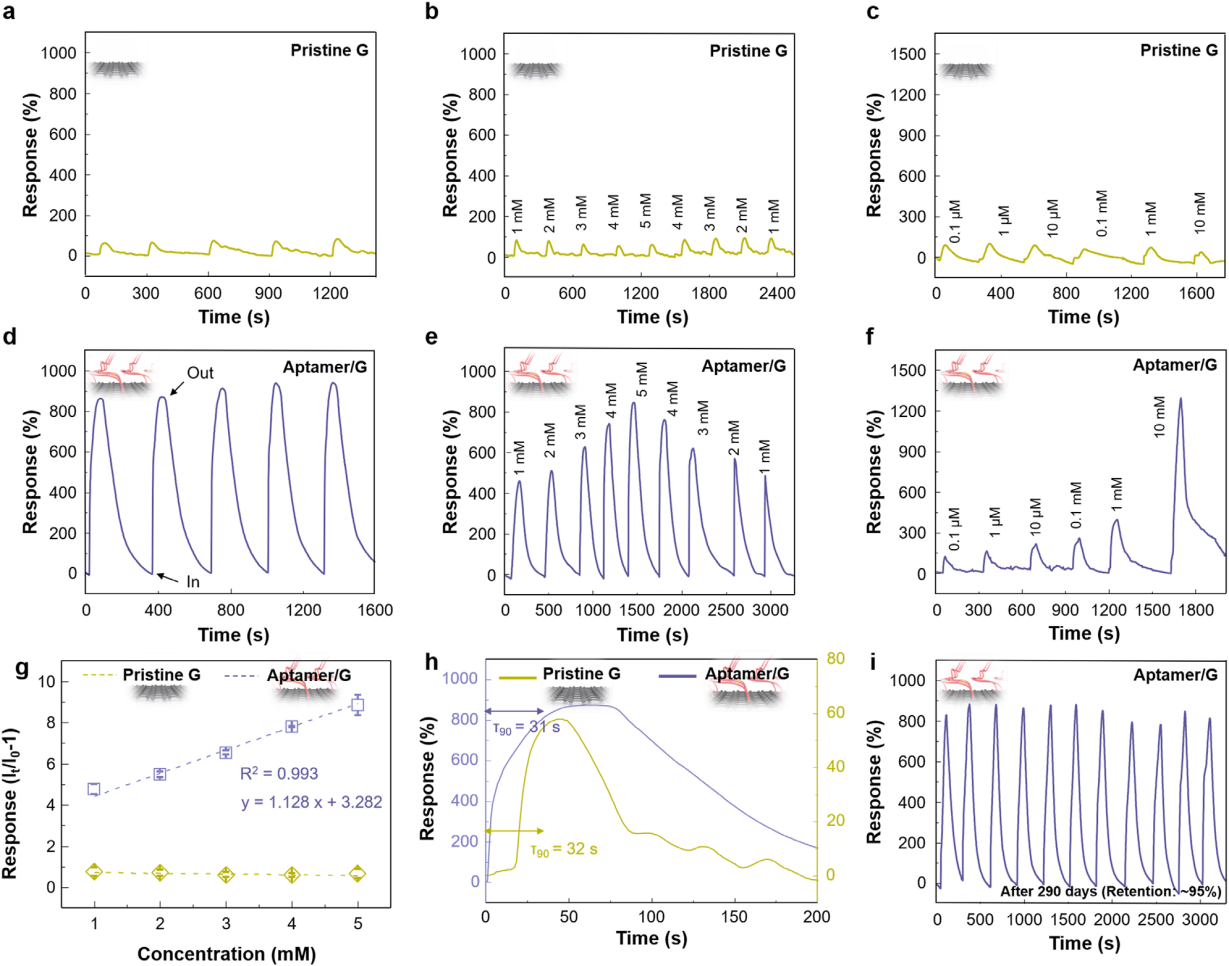
Quantitative sensing performance and selectivity of various graphene‐based quinine sensors. (a) Real‐time response curve of the Pristine G (graphene) sensor to 5 mM quinine. (b) Response of the Pristine G sensor to increasing concentrations of quinine (1–5 mM). (c) Detection limit measurement of the Pristine G sensor. (d) Response profiles of the Aptamer/G (aptamer‐functionalized graphene) sensor to repeated exposure to 5 mM quinine and washing. (e) Response of the Aptamer/G sensor to different concentrations of quinine (1–5 mM). (f) Detection limit measurement of the Aptamer/G sensor. (g) Linearity measurement of sensor response as a function of quinine concentration for Pristine G and Aptamer/G devices. Error bars indicate the standard deviation calculated from measurements of three independent sensor devices (n = 3), showing high consistency with an average RSD of 3.03%. (h) Comparative response kinetics and τ_90_ values of Pristine G and Aptamer/G sensors. The right Y‐axis indicates the magnified response of Pristine G. (i) Long‐term stability of the Aptamer/G sensor. The device retained ∼95% of its initial response magnitude after 290 days. All measurements were performed at a gate voltage of −0.2 V.

To directly benchmark the performance of our device against previously reported quinine sensors, Table 1 summarizes the main characteristics, including detection range, response and recovery time, and reversibility for various quinine sensors. As shown in Table 1, our aptamer‐functionalized graphene FET sensor achieves high sensitivity, rapid signal recovery, and complete reversibility, clearly distinguishing it from conventional electrochemical, fluorescent, and colorimetric sensors [1, 35, 37, 38].

**TABLE 1:** Comparative analysis of other existing quinine detection studies.

Measurement method	Sensing platform	Quinine detection range	Response/recovery time	Reversibility	References
Electrochemical sensor	Split aptamer on gold electrode	0.05 µM–10 µM	∼15 min, N/A	Irreversible	[1]
Fluorescent sensor	J‐aggregating cyanine dye/aptamer	1.5 µM–50 µM	5∼15 min, N/A	Irreversible	[37]
Electrochemical sensor	Aptamer‐invertase conjugate	0.1 µM–10 µM	∼40 min, N/A	Irreversible	[35]
Colorimetric sensor	DNA aptazyme	0.6 µM–37.5 µM	∼30 min, N/A	Irreversible	[38]
FET sensor	Aptamer‐functionalized graphene FET	0.1 µM–10 mM	31 s, ∼200 s	Reversible	This work

### 2.4 Quantitative Assessment, Long‐Term Stability, and Comparative Selectivity Profiling of Functionalized Graphene‐Based Taste Sensors

The detection limits for both devices were determined by testing a broad range of quinine concentrations. While the pristine graphene sensor produced only small, marginally detectable responses at millimolar levels (Figure 4c), the aptamer‐functionalized graphene sensor was able to generate distinct and reliable signals at substantially lower target concentrations (Figure 4f), reflecting its enhanced sensitivity. Quantitative assessment of sensor performance revealed a clear difference between the two systems (Figure 4g) [34]. In these measurements, the pH environment was kept constant to ensure signal reliability and to eliminate potential interference from the intrinsic pH sensitivity of the graphene channel, thereby isolating the target‐specific aptamer‐binding response. To ensure the reliability and batch‐to‐batch reproducibility of the sensing performance, quantitative metrics were derived from measurements conducted on three independent devices (n = 3) under identical conditions. The data are presented as mean values with standard deviations (error bars) in Figure 4g, exhibiting a low average relative standard deviation (RSD) of 3.03% (with a maximum RSD of 5.52% at 5 mM), which confirms the high consistency of the fabrication process. The individual device responses demonstrating this high uniformity are provided in Figure S9. The aptamer‐functionalized graphene taste sensor demonstrated excellent analytical linearity across the examined quinine concentration range, exhibiting a positive response gradient. This enhanced linearity is particularly significant for reliable quantitative sensing applications, as it enables accurate analyte determination through consistent signal to concentration correlation. On the other hand, the graphene‐based taste sensor showed very little reactivity to concentration, and its calibration profile was almost flat, which suggests that its sensing capacity was limited. The superior analytical performance of the aptamer‐functionalized device can be attributed to the specific molecular recognition properties of the MNS‐4.1 aptamer, which facilitates selective binding processes that efficiently modulate the electronic properties of the graphene [1, 8].

The response kinetics analysis revealed that both systems exhibited rapid signal development upon quinine exposure, with τ_90_ values (time to reach 90% of maximum response) of 31 s and 32 s for aptamer‐functionalized graphene and graphene‐based taste sensor, respectively (Figure 4h) [10]. The nearly identical response kinetics observed across both platforms indicates that the overall sensing speed is predominantly governed by interfacial mass transport and analyte diffusion within the microfluidic architecture. Under the controlled flow conditions (0.07 mL/min), the time‐scale for establishing a stable analyte concentration at the graphene‐liquid interface appears to be the rate‐limiting factor, which results in nearly identical response times regardless of surface functionalization. To provide a clearer validation, the response curve for pristine graphene was magnified using an independent Y‐axis scale as shown in Figure 4h. To demonstrate the superior durability of the sensor for practical applications, the long‐term stability was evaluated over an extended period of 290 days. Figure 4i presents the sensor response after prolonged storage. The sensor exhibited outstanding stability, retaining ∼95% of its initial response after 290 days of storage. The calculated degradation rate was exceptionally low at approximately 0.018% per day, indicating that the aptamer‐functionalized graphene surface is highly robust against environmental aging. These results, achieved under ambient conditions, provide indirect evidence of the thermodynamic stability of the aptamer‐functionalized interface, as the device maintained its structural and functional integrity against natural environmental fluctuations over the extended period. Furthermore, the dynamic response transients were analyzed to verify the preservation of signal quality. Figure S10 compares the representative real‐time response curves measured at the initial state and after 290 days. As evidenced by the similar waveform profiles, the signal characteristics, including response kinetics and baseline recovery, remained consistent without significant deterioration. This confirms that the sensor maintains its rapid sensing capability and structural integrity even after prolonged storage [39].

The selectivity profile and molecular recognition mechanism of graphene‐based taste sensors functionalized with Nafion, Au nanoparticles, Calix[4]arene, and aptamers were systematically investigated (Figure 5). While pristine graphene exhibits little analyte differentiation, selective surface modifications establish distinct molecular recognition sites. Nafion coating introduces sulfonate groups with molecular sieving properties, resulting in significantly enhanced H^+^ selectivity; Au nanoparticle decoration enables glucose detection; Nafion/Calix[4]arene composites facilitate clear Na^+^ recognition; and aptamer immobilization provides exclusive quinine selectivity (Figure 5a) [40, 41].

**FIGURE 5:**
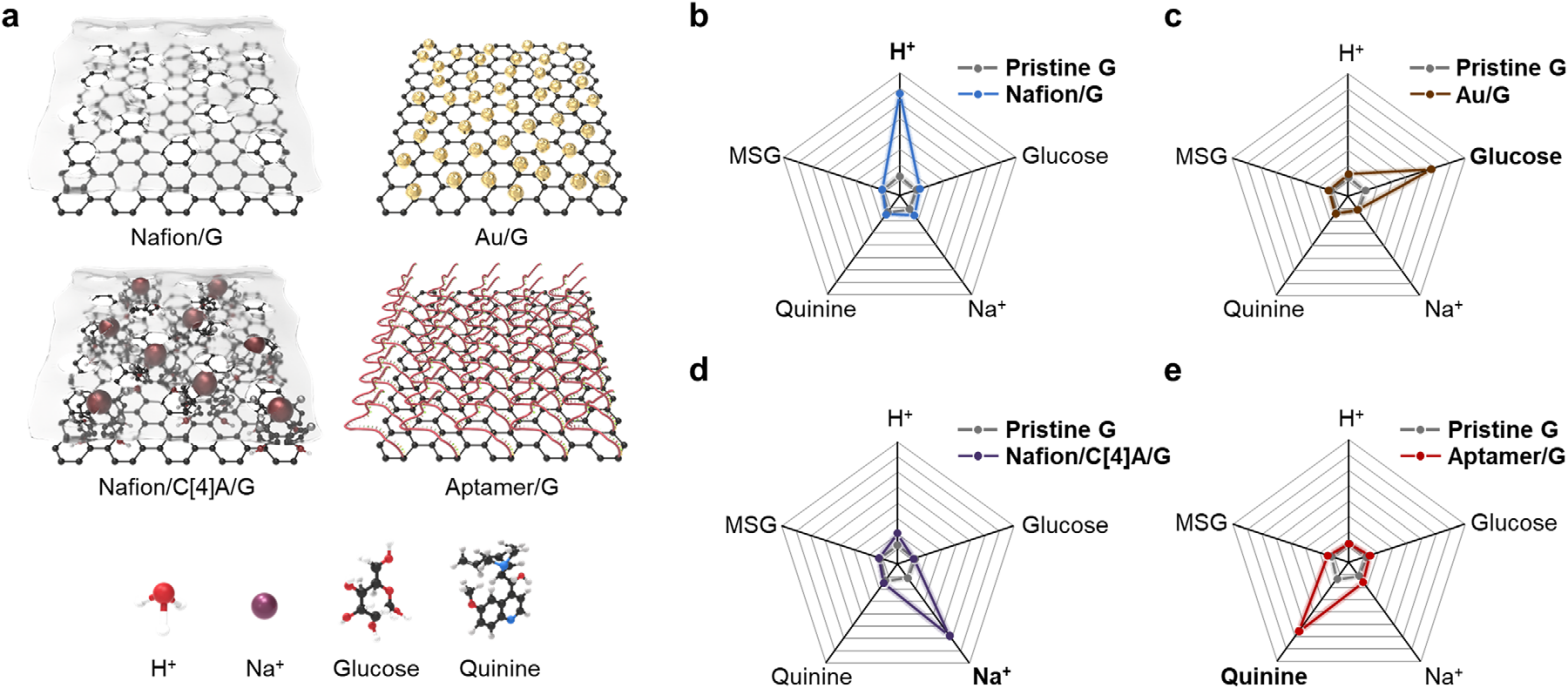
Schematic overview and selectivity mapping of graphene‐based taste sensors. (a) Schematic illustrations of representative taste sensors functionalized with Nafion, Au nanoparticles, Calix[4]arene, or aptamers on graphene (Nafion/G, Au/G, Nafion/C[4]A/G, and Aptamer/G, respectively), and structural representations of the analytes. (b–e) Radar plots showing normalized sensor responses to H^+^, glucose, Na^+^, quinine and MSG: (b) Pristine G (graphene) vs. Nafion/G, (c) Pristine G vs. Au/G, (d) Pristine G vs. Nafion/C[4]A/G, and (e) Pristine G vs. Aptamer/G. The grid represents the sensor response, with each grid interval corresponding to a value of 2. The concentration of all analyte solutions was fixed at 5 mM.

Representative taste components, including H^+^ (sour), glucose (sweet), Na^+^ (salty), quinine (bitter), and MSG (monosodium glutamate, umami) were used at equivalent 5 mM concentrations to compare the selectivity profiles of different graphene‐based taste sensors. Radar plots were adopted for visualizing normalized sensor responses, allowing clear comparison of each device's molecular recognition capabilities (Figure 5b–e). Each radar plot highlights the distinct selectivity fingerprint imparted by each type of surface functionalization: Nafion/G for H^+^, Au/G for glucose, Nafion/C[4]A/G for Na^+^, and Aptamer/G for quinine, with pristine graphene providing minimal differentiation [42, 43]. Furthermore, to address the structural specificity and potential interferences, the sensor's response was validated against a broader range of bitter compounds, including quinidine, cinchonine, cinchonidine, and caffeine (Figure S11). Despite the high degree of structural similarity among the alkaloids, the sensor demonstrated remarkable discrimination for quinine, with negligible cross‐reactivity toward its isomers. This high‐fidelity selectivity, derived from the specific binding pocket of the MNS‐4.1 aptamer, highlights the potential of our graphene‐based platform to be customized for various targets. While the current study focuses on quinine as a model analyte, these results suggest that a comprehensive electronic tongue for broad bitterness sensing could be achieved by integrating an array of different target‐specific aptamers. Additional controls and characterization are presented in Figures S12 (response to 0.1 M HCl) and S13 (FTIR spectra). Further responses are provided for pristine and aptamer‐functionalized graphene (Figures S14, S15) and the other functionalized taste sensors (Figures S16–S18).

### 2.5 Real‐World Validation and Multivariate Taste Discrimination Using Functionalized Graphene‐Based Sensing Systems

The comparative performance of variously functionalized graphene‐based electronic tongue (e‐tongue) systems for selective taste discrimination was evaluated across diverse beverage samples. The experimental setup for real‐world beverage sensing, which consists of sample preparation, loading onto the sensor device, integrated real‐time measurement, LED indicator, and mobile app feedback interface, is depicted in Figure 6a. This comprehensive arrangement highlights the practical workflow for beverage analysis and system integration, demonstrating the applicability of the developed e‐tongue platform for real‐time taste assessment.

**FIGURE 6:**
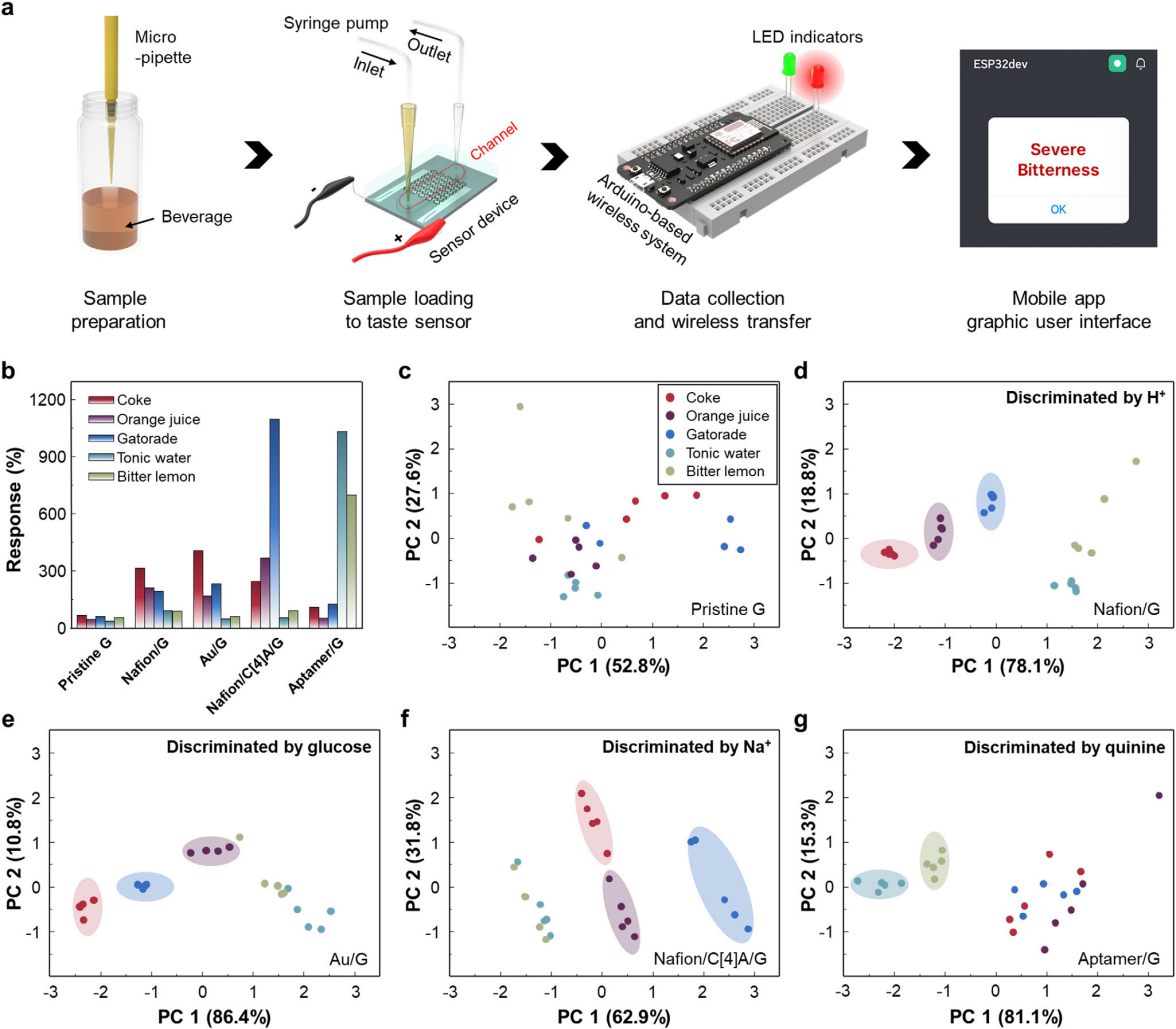
Demonstration of aptamer‐functionalized graphene taste sensor for classification of real beverages. (a) Schematic illustration of taste sensor system. (b) Sensor responses of Pristine G (graphene), Nafion/G, Au/G, Nafion/C[4]A/G (C[4]A = Calix[4]arene), and Aptamer/G devices to various commercial beverage samples (coke, orange juice, Gatorade, tonic water, bitter lemon). (c–g) PCA plots demonstrating beverage discrimination by each sensor: (c) Pristine G, (d) Nafion/G (discriminated by H^+^), (e) Au/G (discriminated by glucose), (f) Nafion/C[4]A/G (discriminated by Na^+^), and (g) Aptamer/G (discriminated by quinine).

The response profiles of five distinct sensing platforms, specifically pristine graphene, Nafion/G, Au/G, Nafion/C[4]A/G, and Aptamer/G, were analyzed upon exposure to common beverages including coke, orange juice, Gatorade, tonic water, and bitter lemon (Figure 6b, Figures S19–S22). The pristine graphene device displayed negligible response differentiation across the beverage samples, exhibiting substantially attenuated signal magnitudes that indicate inherently limited selectivity for complex analyte mixtures. In contrast, the functionalized graphene systems showed noticeably higher signal intensities with distinctive selectivity profiles directly correlated with their surface chemical modifications [44].

Principal component analysis (PCA) was employed to evaluate the discriminatory capabilities of each sensing platform. In the PCA plots (Figure 6c–g), translucent shaded ellipses were added as visual guides to indicate the boundaries of sample clusters. The unfunctionalized graphene‐based taste sensor exhibited poor classification performance between different beverages (Figure 6c). This limited discrimination can be attributed to the non‐specific interactions between graphene and beverage components in the absence of selective recognition elements [7, 45]. The functionalized systems demonstrated remarkable improvements in taste discrimination, each with distinctive selectivity patterns aligned with their molecular recognition capabilities. The Nafion/G taste sensor effectively distinguished beverages based on their H^+^ (sourness) concentration, producing clearly separated groups that enable identification of beverages with varying sourness profiles (Figure 6d). Similarly, the Au/G taste sensor demonstrated exceptional glucose (sweetness) discrimination capabilities (Figure 6e), with well‐defined separation between beverage classes. The Nafion/C[4]A/G taste sensor exhibited selective sodium ion (Na^+^, saltiness) recognition (Figure 6f), effectively discriminating beverages. Most notably, the Aptamer/G taste sensor demonstrated superior performance in quinine (bitterness) detection (Figure 6g), with particularly clear segregation of beverages having a bitter taste [46]. The PCA plot reveals distinctive clustering of tonic water and bitter lemon samples, both known for their characteristic bitter taste profiles derived from quinine content. This exceptional bitterness discrimination capability can be attributed to the specific molecular recognition properties of the quinine‐binding aptamer, which undergoes conformational changes upon target binding that modulate the electrical properties of the graphene channel [10]. To quantitatively validate this separation, Silhouette Scores were calculated for each sensor. All functionalized sensors exhibited high scores exceeding 0.5 (Nafion/G: 0.80, Au/G: 0.83, Nafion/C[4]A/G: 0.58, Aptamer/G: 0.68), statistically confirming that the beverage clusters are distinct and well‐separated [47].

For selective taste recognition, surface modification is essential, as evidenced by the distinct performance differences observed among the various functionalized graphene surfaces. By integrating specific molecular recognition elements with the remarkable electrical properties of graphene, a tunable sensing platform can be developed that enables the identification of complex beverage compositions based on their fundamental taste components. Furthermore, the successful implementation of the sensing system with Arduino‐based signal processing and mobile connectivity demonstrates the potential for real‐world deployment in beverage quality control, authentication, and freshness monitoring applications [48]. The developed multifunctional e‐tongue array represents a significant advancement toward electronic taste sensing systems with human‐like discrimination capabilities combined with the precision and objectivity of instrumental analysis.

## 3 Conclusions

In this work, we developed and validated an aptamer‐functionalized graphene taste sensor capable of highly selective and reversible quinine detection. Through systematic material characterization and device engineering, we demonstrated that non‐covalent aptamer immobilization on graphene enables specific molecular recognition, with target‐induced conformational changes directly modulating the local charge environment and electronic properties of the sensor. The platform exhibited reliable signal reversibility, high sensitivity, and remarkable long‐term stability, even after extended storage and repeated use. By combining this advanced quinine sensor with our existing platforms, we finally realized a high‐performance e‐tongue system that exhibited clear selectivity during real beverage demonstrations. Comparative studies with other functionalized graphene sensors confirmed the superior selectivity and analytical performance of the aptamer‐functionalized graphene system, particularly for bitterness profiling in complex beverage samples. Integration with microfluidic delivery, real‐time signal processing, and mobile connectivity further highlights its practical potential for advanced taste sensing and beverage quality monitoring. Overall, this study establishes a flexible framework for the design of next‐generation biosensors that bridge molecular specificity and electronic transduction provided by nanomaterials for real‐world analytical applications.

## 4 Experimental Section

### 4.1 Fabrication of IDEs and Microfluidic Channel

Photolithography patterning and e‐beam evaporation of Pt/Ti (100/30 nm) onto SiO_2_/Si substrates with a SiO_2_ thickness of 300 nm were used to create interdigitated electrodes (IDEs). The patterned electrodes were made with a gap distance of 5 µm and a dimension of 1 mm × 1 mm. After the patterning process, the IDE substrates were cleaned using sonication for 20 min with acetone and isopropanol (IPA), and then N_2_ gas was used to dry them. To produce microfluidic channels, a 4‐inch SiO_2_/Si wafer was first cleaned by sonication in acetone and IPA for 20 min, and then it was dried under N_2_ flow. An adhesive layer was created by spin‐coating SU‐8 2 at 3000 rpm for 30 s, then soft baking (65°C for 2 min, 95°C for 2 min), UV exposure (2 min), and hard baking (180°C for 3 min). The SU‐8 50 was spin‐coated for 30 s at 2000 rpm for the photoresist (PR) layer. 5 h of soft baking were done at 65°C and 5 h at 95°C. 3 min of hard baking at 180°C came after 30 s of UV irradiation. After 3 min of development, a DI rinse and N_2_ gas drying were finished. The curing agent (Sylgard 184) and PDMS monomer were combined in a 10:1 weight ratio. In a vacuum chamber, the prepared PDMS was degassed after being poured over an SU‐8 template. Following 1 h of curing process at 80°C, the patterned microfluidic channel was sliced with a razor and removed [6, 14].

### 4.2 Device Manufacturing Process

Quinine aptamer (sequence: 5’‐GGG AGA CAA GGA TAA ATC CTT CAA TGA AGT GGG TCG ACA‐3’ / 39‐mer) was purchased from both IDT (Integrated DNA Technologies) and Bioneer. DEPC‐treated water was purchased from Biosesang, Eva Green dye (20 X) was purchased from Biofact. In all experiments, aptamers were diluted to a concentration 1 mM with DEPC‐treated water.

High purity copper foil (99.99%) was used in a chemical vapor deposition (CVD) process to create graphene layer at 1000°C with a hydrocarbon source (CH_4_, 35 sccm) and hydrogen (H_2_, 70 sccm) at 0.180 Torr. Poly [methyl methacrylate] (PMMA, 50 mg/mL in chlorobenzene) was spin‐coated on the graphene‐coated copper foil after CVD‐graphene was cut into the shape of the IDE substrate. The baking procedure was then carried out for 15 min at 60°C. The other side of the Cu foil was then etched by letting it float in a 0.2 M ammonium persulfate solution for 2 h. Onto an IDE substrate, the graphene was transferred. Following a 24 h drying period at 50°C in an oven, the PMMA layer was eliminated with acetone at 60°C for 15 min [21].

The aptamer‐functionalized graphene sample was prepared by sequentially applying 5 drops of 2 µL quinine aptamer solution, with a 3 h drying interval between each drop to ensure complete surface coverage. For the Nafion/G sample, Nafion 117 solution was spin‐coated onto the graphene layer at 2000 rpm for 30 s. Prior to spin coating, source and drain electrodes were protected with tape to prevent contamination. The Nafion/G sample was then dried at 50°C for 30 min. The Au/G sample was fabricated by depositing 2 nm gold nanoparticles using an e‐beam evaporator. For the Nafion/C[4]A/G sample, a 5 mM Calix[4]arene solution dispersed in N,N‐dimethylmethanamide was applied in ten sequential drops of 2 µL each. A 3 min drying time between drops was maintained to ensure complete coverage of the graphene surface. Nafion was spin‐coated utilizing the previously described technique after Calix[4]arene deposition. The microfluidic channel graphene‐based taste sensor was produced by attaching the channel‐patterned PDMS to the graphene‐based taste sensor samples. To stabilize the adhesion between the IDE and channel‐patterned PDMS, O_2_ plasma was applied to the PDMS with acrylic transfer tape (Samchun Chemicals) for 120 s at a plasma power of 125 W and an O_2_ flow rate of 60 sccm. The Cu wires were placed on the source and drain electrodes, and PDMS was connected [6, 14].

### 4.3 Solution Preparation and Analysis Methods

Quinine powder (Sigma‐Aldrich, 98%) was dissolved in 0.1 M HCl to prepare quinine solutions with concentrations ranging from 100 nM to 10 mM. We diluted HCl (Daejung, 35.4%) solutions to yield concentration of 5 mM. NaCl (Daejung, 99.5%), MSG (Sigma‐Aldrich, 99%), KCl (Daejung, 99%), CaCl_2_ (Sigma‐Aldrich, 97%), MgCl_2_ (Sigma‐Aldrich, 98%), NH_4_Cl (Junsei, 99.5%), citric acid (Daejung, 99.5%), ascorbic acid (Junsei, 99.6%), uric acid (Sigma‐Aldrich, 99%), and glutamic acid (Sigma‐Aldrich, 99%) powders were dissolved in DI water to prepare solutions with a concentration 5 mM. A vial containing the carbonated drinks was filled and covered with parafilm. Holes were punched in the parafilm cover, which was left in place for a few days in order to extract the carbonic acid gas from the beverage. The Nafion 117 solution was purchased from Sigma‐Aldrich. The TEM image and SAED pattern of the graphene layer were acquired from TEM (JEOL, JEM‐2100F). The chemical bonding profile was analyzed using XPS (ULVAC‐PHI, VersaProbe III). A Raman spectrometer (HORIBA, LabRAM HR Evolution) was used to demonstrate the existence of the graphene layer. The fluorescence intensity was measured using the Azure 600 imaging system from Azure Biosystems. The Sybr Green channel was used to measure the fluorescence of Eva Green dye.

### 4.4 Measurement System and Characterization

Two syringe pumps (Pump 11 Elite Infusion/Withdrawal Programmable Dual Syringe, Figure S23) were used to infuse and withdraw the base solution. Photographs and a video demonstration of the integrated sensing platform are provided in Figure S24 and Supplemental Video 1, respectively. When the target solution (100 µL) was injected using a pipette, the infusion pump was stopped (while the withdrawal pump remained running), and the flow rate was set to 0.07 mL/min. The optimization of the flow rate was imperative to balance sensor stability with response kinetics. This gentle flow condition minimizes the shear‐induced desorption of the π‐π stacked aptamers to ensure baseline stability, while still providing sufficient mass transport for reversible sensor operation. Following the injection of the target solution, the infusion pump was turned back on for 100 s. The cycle time for each solution was 300 s. A Pt wire was electrically connected to the back‑gate contact of the silicon substrate to apply the gate voltage in the bottom‑gate configuration. The electrical characteristics of the device were evaluated using an Agilent 4156 C semiconductor analyzer with a gate voltage of −0.2 V and a source‐drain voltage of 0.1 V. The sensor response was calculated using the following equation [6]:

(2) $$\mathrm{Response}\;\;\left(\%\right)=\frac{I_{t}-I_{0}}{I_{0}}\;\times \;100\;\%\;=\frac{\Delta I}{I_{0}}\;\times \;100\;\%$$

where *I_0_* is the baseline current and *I_t_* is the current after analyte exposure.

## Funding

This work was supported by the Ministry of Science and ICT, South Korea (RS‐2024‐00405016, RS‐2026‐25589527, 1.250021.01, and RS‐2024‐00421181), and the National Research Council of Science & Technology (NST), funded by the Korean government (GTL25021‐230).

## Conflicts of Interest

Ho Won Jang is a member of the *Exploration* editorial board, and he was not involved in the handling or peer review process of this manuscript. The authors declare no conflict of interest.

## AI Statement

ChatGPT and Codex (OpenAI) were used for language editing and textual refinement of the main manuscript (including figure captions), Supporting Information, and graphical abstract text. Limited assistance was also obtained for PCA coding, Arduino implementation, and preparation of the mobile‐interface graphic within Figure 6a. The authors reviewed and verified the AI‐assisted outputs and take full responsibility for the manuscript.

## Supporting information




**Supporting File 1**: exp270232‐sup‐0001‐VideoS1.mp4.

**Supporting File 2**: exp270232‐sup‐0002‐SuppMat.docx.
