# Supplementary material for: High‐Performance and Reversible Bitter Taste Sensing Using Aptamer‐Functionalized Graphene Channel for Real‐Time Electronic Tongue
Source: Exploration (Beijing). 2026 Oct 27;6(5):70232. doi: 10.1002/exp2.70232 (PMC13710939; doi:10.1002/exp2.70232)
Supplement: Supplementary file 1 [file EXP2-6-70232-s001.docx]

Supporting Information

**High-Performance and Reversible Bitter Taste Sensing using Aptamer-Functionalized Graphene Channel for Real-Time Electronic Tongue**

**Figure S1.** Publication numbers for sensors targeting key taste substances (pH, glucose, Na^+^, quinine).

**Figure S2.** Schematic illustration of MNS-4.1 aptamer structure with detailed nucleotide sequence.

**Figure S3.** (a) Photograph of the Azure 600 imaging system used for fluorescence detection. (b) Schematic illustration of the fluorescence activation mechanism of Eva Green upon binding to DNA.

| Name | Sequence (5' to 3') | Length |
| --- | --- | --- |
| MNS-4.1 | GGG AGA CAA GGA TAA ATC CTT CAA TGA AGT GGG TCG ACA | 39-mer |
| Poly-T DNA | TTT TTT TTT TTT TTT TTT TTT TTT TTT TTT TTT TTT TTT | 39-mer |
| Scrambled DNA | GTA AGG ACT GAT AGG ACG TAT CAG AAC TGG AGT GAC AAC | 39-mer |

**Figure S4.** Specificity test of the graphene-based sensor using control DNA sequences. The DNA sequences of the native MNS-4.1 aptamer and the control sequences (Poly-T and Scrambled) used for validation are listed in the table (top). Note that the scrambled aptamer possesses an identical nucleotide composition to MNS-4.1 but with a randomized order. (a, b) Real-time response transients of the (a) poly-T DNA/graphene and (b) scrambled DNA/graphene sensors upon exposure to 5 mM quinine solution. The negligible responses in both controls confirm that the sensing signal originates from the specific conformational change of the MNS-4.1 aptamer.

**Figure S5.** Optical microscopy images of (a) graphene surface and (b) aptamer-functionalized graphene surface after transfer onto the substrate for selective quinine detection.

**Figure S6.** Schematic illustration of the device structure and electrical measurement configuration, highlighting the detection site on the SiO_2_/Si substrate.

**Figure S7.** TEM image (a) and SAED patterns (b) confirming morphology of the graphene layer.

**Figure S8.** Statistical analysis of the monolayer graphene lattice features via Raman spectroscopic mapping. (a) Raman intensity map for the I_2D_/I_G_ ratio acquired from the graphene channel area, demonstrating long-range spatial uniformity. (b) Statistical histogram of the I_2D_/I_G_ ratio with a distinct peak centered at ≥ 2, providing definitive proof for the predominant formation of high-quality monolayer graphene across the large-scale substrate.

**Figure S9.** Device-to-device reproducibility test. Comparison of the dynamic sensing responses from three independent devices (n = 3) for (a-c) Pristine Graphene (Pristine G) and (d-f) Aptamer-functionalized Graphene (Aptamer/G) sensors upon exposure to 5 mM quinine. The consistent response magnitudes and kinetics observed across different devices demonstrate the high uniformity and reliability of the sensor fabrication process. The error bars presented in Figure 4g of the main manuscript were calculated based on these datasets.

**Figure S10.** Comparison of the representative dynamic response transients of the sensor. The graph displays the normalized response curves measured at the initial state (solid line) and after 290 days of storage (dotted line). The consistent signal patterns and recovery characteristics confirm that the sensor maintains its kinetic stability even after long-term storage.

**Figure S11.** Comparative response analysis of pristine and aptamer-functionalized graphene sensors toward various alkaloids. Real-time response curves of (a–d) the pristine graphene device and (e–h) the aptamer-functionalized graphene device upon exposure to 5 mM of quinidine, cinchonine, cinchonidine, and caffeine.

**Figure S12.** Repetitive test of aptamer/graphene-based taste sensor using exposures to 0.1 M HCl solution.

**Figure S13.** FTIR spectra of aptamer, aptamer/graphene (aptamer-functionalized graphene) samples.

**Figure S14.** Repetitive response of the graphene-based taste sensor to various ions and small molecules at 5 mM concentration. (a) HCl, (b) KCl, (c) NaCl, (d) glucose, (e) monosodium glutamate (MSG), (f) CaCl_2_, (g) MgCl_2_, (h) NH_4_Cl, (i) citric acid, (j) ascorbic acid, (k) uric acid, and (l) glutamic acid.

**Figure S15.** Repetitive response of the aptamer/graphene-based taste sensor to various ions and small molecules at 5 mM concentration. (a) HCl, (b) KCl, (c) NaCl, (d) glucose, (e) MSG, (f) CaCl_2_, (g) MgCl_2_, (h) NH_4_Cl, (i) citric acid, (j) ascorbic acid, (k) uric acid, and (l) glutamic acid.

**Figure S16.** Repetitive response of the Nafion/graphene-based taste sensor to taste factors at 5 mM concentration: (a) HCl, (b) glucose, (c) NaCl, (d) quinine, and (e) MSG.

**Figure S17.** Repetitive response of the Au/graphene-based taste sensor to taste factors at 5 mM concentration: (a) HCl, (b) glucose, (c) NaCl, (d) quinine, and (e) MSG.

**Figure S18.** Repetitive response of the Nafion/Calix[4]arene/graphene-based taste sensor to taste factors at 5 mM concentration: (a) HCl, (b) glucose, (c) NaCl, (d) quinine, and (e) MSG.

**Figure S19.** Repetitive response of the Nafion/graphene-based taste sensor to various soft drinks: (a) Coke, (b) orange juice, (c) Gatorade, (d) tonic water, and (e) bitter lemon.

**Figure S20**. Repetitive response of the Au/graphene-based taste sensor to various soft drinks: (a) Coke, (b) orange juice, (c) Gatorade, (d) tonic water, and (e) bitter lemon.

**Figure S21.** Repetitive response of the Nafion/Calix[4]arene/graphene-based taste sensor to various soft drinks: (a) Coke, (b) orange juice, (c) Gatorade, (d) tonic water, and (e) bitter lemon.

**Figure S22.** Repetitive response of the aptamer/graphene-based taste sensor to various soft drinks: (a) Coke, (b) orange juice, (c) Gatorade, (d) tonic water, and (e) bitter lemon.

**Figure S23.** Representative images of the microfluidic measurement setup, showing a dual syringe pump system for controlled solution delivery and a microfluidic PDMS channel with pipette tips positioned on the sensor substrate.

**Figure S24.** Representative images of the integrated sensing platform for beverage analysis, showing (from left) sample preparation, sensor device operation, electronic signal processing with Arduino and LEDs, and real-time notification via mobile app.
